# Synthesis of Pyridine-Dicarboxamide-Cyclohexanone Derivatives: Anticancer and α-Glucosidase Inhibitory Activities and In Silico Study

**DOI:** 10.3390/molecules24071332

**Published:** 2019-04-04

**Authors:** Abdullah Mohammed Al-Majid, Mohammad Shahidul Islam, Saleh Atef, Fardous F. El-Senduny, Farid A. Badria, Yaseen A. M. M. Elshaier, M. Ali, Assem Barakat, A. F. M. Motiur Rahman

**Affiliations:** 1 Department of Chemistry, College of Science, King Saud University, P. O. Box 2455, Riyadh 11451, Saudi Arabia; amajid@ksu.edu.sa (A.M.A.-M.); shahid.10amui@gmail.com (M.S.I.); aboatef2008@gmail.com (S.A.); maly.c@ksu.edu.sa (M.A.); 2 Department of Chemistry, Faculty of Science, Mansura University, Mansura 35516, Egypt; biobotany@gmail.com; 3 Department of Pharmacognosy, Faculty of Pharmacy, Mansoura University, Mansoura 35516, Egypt; faridbadria@gmail.com; 4 Department of Organic and Medicinal chemistry, Faculty of Pharmacy, University of Sadat City, Menofia 32958, Egypt; yaseen.elshaier@fop.usc.edu.eg; 5 Department of Chemistry, Faculty of Science, Alexandria University, P.O. Box 426, Ibrahimia, Alexandria 21321, Egypt; 6 Department of Pharmaceutical Chemistry, College of Pharmacy, King Saud University, Riyadh 11451, Saudi Arabia; afmrahman@ksu.edu.sa

**Keywords:** malonamide, Michael addition reaction, cytotoxicity, cancer, α-glucosidase, docking

## Abstract

An efficient and practical method for the synthesis of 2,6-diaryl-4-oxo-*N*,*N*′-di(pyridin-2-yl)cyclohexane-1,1-dicarboxamide is described in this present study, which occurs through a double Michael addition reaction between diamide and various dibenzalacetones. The reaction was carried out in dichloromethane (DCM) in the presence of 1,8-diazabicyclo[5.4.0]undec-7-ene (DBU). The anticancer activities of the synthesized compounds were evaluated in several cancer cell lines, including MCF-7, MDA-MB-231, SAS, PC-3, HCT-116, HuH-7 and HepG2 cells. From these experiments, we determined that MDA-MB-231 was the most sensitive cancer cell line to the compounds **3c**, **3e**, **3d**, **3j** and **3l**, which exhibited variable anticancer activities (**3l** [IC_50_ = 5 ± 0.25 µM] > 3e [IC_50_ = 5 ± 0.5 µM] > **3c** [IC_50_ = 7 ± 1.12 µM] > **3d** [IC_50_ = 18 ± 0.87 µM] > **3j** [IC_50_ = 45 ± 3 µM]). Of these, **3l** (substituted *p*-trifluoromethylphenyl and chloropyridine) showed good potency (IC_50_ = 6 ± 0.78 µM) against HCT-116 colorectal cancer cells and exhibited high toxicity against HuH-7 liver cancer cells (IC_50_ = 4.5 ± 0.3 µM). These values were three times higher than the values reported for cisplatin (IC_50_ of 8 ± 0.76 and 14.7 ± 0.5 µM against HCT-116 and HuH-7 cells, respectively). The highest α-glucosidase inhibitory activity was detected for the **3d**, **3i** and **3j** compounds. The details of the binding mode of the active compounds were clarified by molecular docking studies.

## 1. Introduction

**Figure 1:**
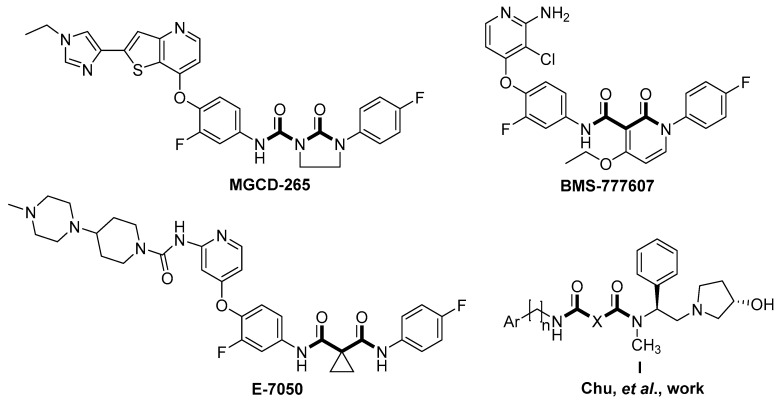
Structures of some biologically active *N*,*N*′-malonamide derivatives.

Several malonamide-based anticancer agents with promising cytotoxic activities have been identified from natural and synthetic sources [1,2]. For instance, golvatinib (E-7050) is a clinical agent with dual inhibitory activity against c-Met and vascular endothelial growth factor receptor-2 (VEGFR-2) tyrosine kinases and is known to exhibit high antineoplastic potential [3] (Figure 1). BMS-777607, one of the malonamide-based molecules with Met inhibition activity, has entered phase II clinical trials [4,5,6,7]. Chu et al. provided a malonamide-based small molecule **I**, which is thought to be effective as a selective κ optical receptor agonist [8]. Our research team recently developed several malonamide motifs as α-glucosidase inhibitory agents [9,10], which have moderate cytotoxicity against HeLa, H460, MCF-7 and 3T3 cell lines [9].

Functionalized cyclohexanones that utilizes stereogenic centers as valuable building blocks are known to be present at the core of several natural products and drug candidates. Functionalized cyclohexanones are embedded in the antidepressant and dissociative anesthetic drugs Ketanest^®^S [11] and Vasoxyl^®^ methoxamine (for the treatment of hypotension) [12]. These molecules possess antibacterial [13], anticonvulsant [14], antifungal and anticancer [15] properties. In general, cyclohexanone is a common scaffold in various bioactive heterocycles of medicinal interests, particularly those used for the treatment of asthma and central nervous system (CNS)- and chronic obstructive pulmonary diseases (COPD)-related diseases, due to its inhibitory activity against phosphodiesterase 4 (PDE4) [16,17]. 

As a continuation of our search for malonamide-based potent anticancer agents, in this present study, we demonstrate the preparation of a new library of malonamide-based compounds (**3a**–**m**) through the incorporation of important scaffolds, namely cyclohexanone and dicarboximide derivatives, in a single molecule and highlight their anticancer and α-glucosidase inhibitory activities.

## 2. Results

### 2.1. Synthesis of ***3a–m***

**Scheme 1:**
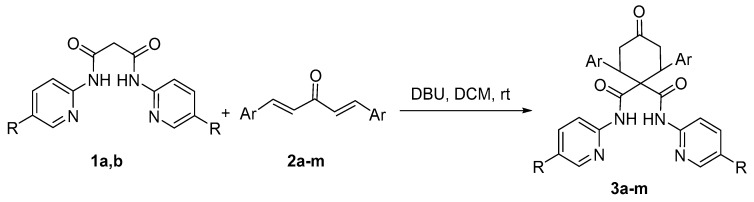
The synthesis of the target compound **3a**–**m**.

Anticancer compounds incorporating 2,6-diaryl-4-oxo-*N*,*N*′-di(pyridin-2-yl)cyclohexane-1,1-dicarboxamide **3a**–**m** via a double Michael addition reaction were prepared according to the previously described method [8,9]. The reaction was carried out by mixing diamide **1a**,**b** that was carrying an active methylene group with dienone **2a**–**m** (Scheme 1, Table 1) in dichloromethane (DCM) at room temperature (24 °C) for 2–3 h. The process was carried out in the presence of DBU (1,8-Diazabicyclo[5.4.0]undec-7-ene) to obtain the final compound **3a**–**m** at an acceptable yield (33–89%). The chemical structures of the Michael-adducts were deduced with infrared (IR) spectroscopy, mass spectrometry (MS), ^1^H-nuclear magnetic resonance (NMR), ^13^C-NMR and elemental analysis (CHN).

### 2.2. Biological Activities

#### 2.2.1. Anticancer Activity

To evaluate the anticancer activity of the 13 newly synthesized compounds, we screened their activities at a concentration of 50 μM against seven cancer cell lines, including breast cancer (positive [MCF-7] and negative [MDA-MB-231] for estrogen receptor expression), tongue carcinoma (SAS), prostate cancer (PC-3), colorectal cancer (HCT-116) and liver cancer (HuH-7 and HepG2) cell lines. The results revealed that only five compounds (**3c**, **3d**, **3e**, **3k** and **3l**) showed different levels of anticancer activities (Table 2). The 3-(4,5-dimethylthiazol-2-yl)-2,5-diphenyltetrazolium bromide (MTT) assay was performed to determine the concentration of the active compounds needed to kill 50% of cells. The results revealed that the compound **3c** (substituted *p*-chlorophenyl) killed 50% of ER-negative breast cancer cells (IC_50_ = 7 ± 1.12 μM) and HepG2 (IC_50_ = 8 ± 0.89 μM) at concentrations lower than that of the common chemotherapeutic drug cisplatin (IC_50_ = 15 ± 0.71 and 10 ± 0.65 μM against ER-negative breast cancer cells and HepG2, respectively). Furthermore, the anticancer activities of the compounds **3e** (substituted *p*-bromophenyl, IC_50_ = 5 ± 0.5 μM) and **3l** (substituted *p*-trifluoromethylphenyl and chloropyridine, IC_50_ = 5 ± 0.25 μM) were stronger than the activity of cisplatin (IC_50_ = 15 ± 0.71 μM) against MDA-MB-231 cells. The compounds **3c**, **3e** and **3l** also exhibited moderate anticancer activities against the ER-positive MCF-7 cell lines (IC_50_ = 10 ± 0.62, 12 ± 0.54 and 18 ± 1.71 μM, respectively). Only two compounds (**3c** and **3l**) exhibited moderate anticancer activities against the tongue carcinoma cell line (SAS, IC_50_ = 15 ± 1.3 and 9 ± 0.38 μM, respectively). Of these molecules, the compound **3l** (substituted *p*-trifluoromethylphenyl and chloropyridine) showed good potency (IC_50_ = 6 ± 0.78 µM) against HCT-116 colorectal cancer cells and exhibited high efficacy against HuH-7 liver cancer cells (IC_50_ = 4.5 ± 0.3 µM). These values were three times higher than the values reported for cisplatin (IC_50_ of 8 ± 0.76 and 14.7 ± 0.5 µM against HCT-116 and HuH-7 cells, respectively) (Table 2).

#### 2.2.2. α-Glucosidase Inhibitory Activity

The synthesized compounds were screened for their ability to inhibit α-glucosidase activity and the results are summarized in Table 3. Among all the compounds, **3d**, **3i** and **3j** exhibited excellent α-glucosidase inhibitory activities while the rest of the compounds were inactive. 

However, the compound **3j** (substituted furan and chloropyridine moieties) showed the highest α-glucosidase inhibitory activity with an IC_50_ value of 124.24 ± 0.16 μmol/L, followed by the compounds **3d** (substituted 2,4-dichlorobenzene and pyridine moieties; IC_50_ of 148.18 ± 3.02 μmol/L) and **3i** (substituted thiophene and chloropyridine moieties; IC_50_ of 418.21 ± 1.02 μmol/L). Acarbose was used as a standard control (IC_50_ = 32.71 ± 1.17 μmol/L).

### 2.3. Molecular Docking Study

As evident from the data represented in Table 1, the synthesized compounds exhibited diversity in their anticancer activities and only compounds **3l**, **3c** and **3e** exerted strong anticancer activities. We subsequently investigated the protein that interacts with these three compounds in a unique binding mode and exhibits strong binding interactions in a manner different from those with the inactive analogues. Docking procedures were performed in the presence of different proteins, including tyrosine kinase (ID: 3F82 [6], mammalian target of rapamycin (mTOR; ID: 4JSV) [18], epidermal growth factor receptor (EGFR; ID: 1M17) [19] and extracellular signal-regulated kinase (ERK; ID: 2OJG) [20] and 2OJJ [20,21,22,23].

**Figure 2:**
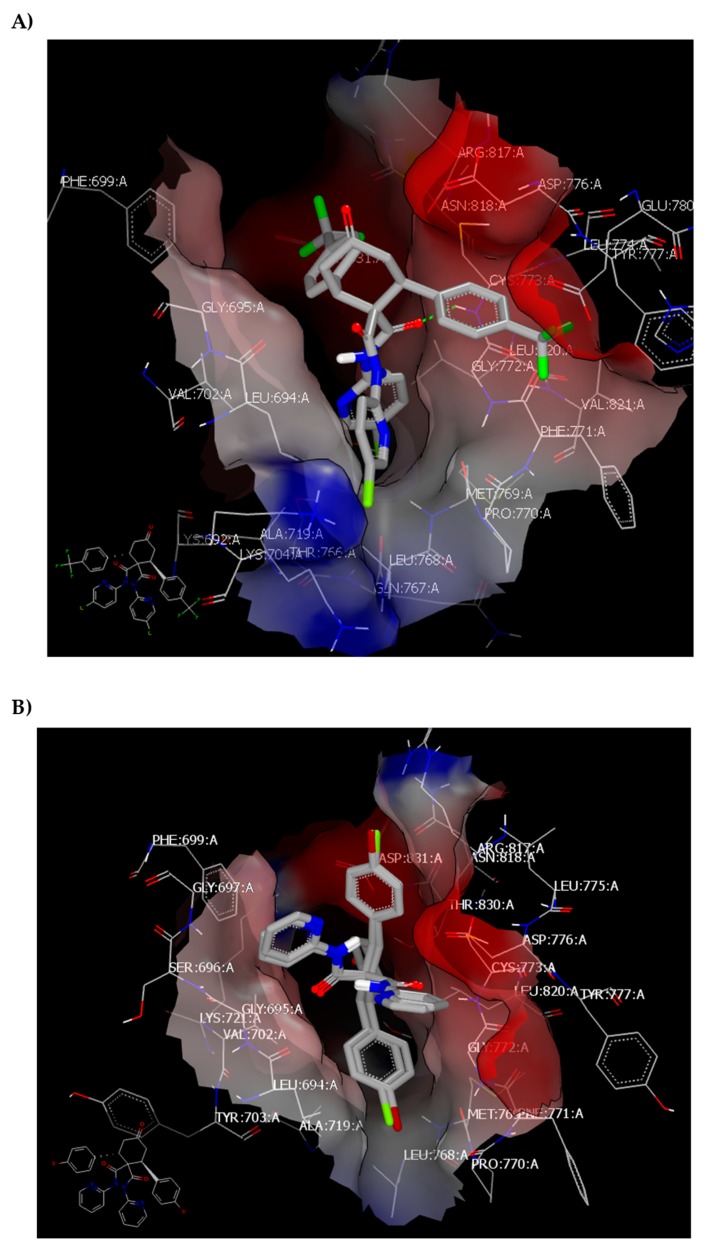
(**A**) Snapshot of **3l** in ID:1M7 showing the HB interaction with Gly 772; (**B**) Snapshot of **3c** and **3e** (ID:1M17) overlaid on each other to show the hydrophobic–hydrophobic interactions.

We found that the active compounds docked well with EGFR and showed a specific strong interaction pattern. The compound **3l** formed HB (acceptor) with the oxygen of amidic carbonyl of the amino acid residue Gly 772 AA, (Figure 2A). This amino acid interacts with the standard erlotinib in a non-HB manner [19]. Both the compounds **3e** and **3c** showed similar behavior in terms of binding mode and docking pose with the receptor through hydrophobic–hydrophobic interactions (Figure 2B). The inactive analogs, such as the compound **3m**, showed a different binding interaction in comparison to **3c** and **3e**. 

### 2.4. Structure–Activity Relationship (SAR)

The reason underlying the potent activity of only those three compounds was further investigated. The analysis showed that there is a similarity in the three-dimensional shape and electrostatic potential of those three compounds [24]. Shape similarity (3D similarity) is considered to be a fundamental descriptor for computational drug discovery and is an important characteristic to correctly model and accurately understand the protein–ligand interaction. The shape provides information on neighborhood behavior and the high similarity in shape is reflective of the consistent biological properties [25].

The final compounds contained four aromatic rings as the substituents of the cyclohexanone ring (3, 4, 4, 5), which indicates its highly lipophilic nature that may facilitate the efflux of drugs outside the cells and subsequently decrease the activity. 

Based on the docking results for all final compounds, it was found that the pyridine carboxamides and the para-substituent in the phenyl ring linked to the cyclohexanone ring determine the geometry of each compound (3D structure) and reflect the orientation of each scaffold in the side of the receptor clefts. The presence of the dipyridine carboxamide skeleton is essential for the activity of the compound. The substitution of pyridine ring is not important, while the para-substitution with an electron-withdrawing group (except fluorine) on the aromatic moiety is essential.

## 3. Materials and Methods 

### 3.1. Experimental

General procedure (GP): Dienones **2a**–**m** (0.25 mmol) and diamide **1a** or **1b** (74 mg, 0.25 mmol) were dissolved in 10 mL of dry CH_2_Cl_2_ in a 25 mL round bottom flask. DBU (3 eq, 114 mg, 0.75 mmol) was added to the reaction, which was subsequently stirred for 2–3 h. After the reaction was completed as determined by TLC, the crude material was subjected to column chromatography using ethyl acetate/*n*-hexane (2:3) to give the desired compounds **3a**–**m**.

4-Oxo-2,6-diphenyl-*N*,*N*′-di(pyridin-2-yl)cyclohexane-1,1-dicarboxamide (**3a**). Yield 120 mg (0.22 mmol, 89%); m.p. 248–249 °C; ^1^H-NMR (DMSO-*d*_6_, 400 MHz) δ: 2.35 and 2.37 (dd, 2H, *J* = 4.4 Hz and 11.6 Hz, CH_2_), 2.69 (t, 2H, *J* = 12.0 Hz, CH), 3.99 and 4.01 (dd, 2H, *J* = 4.8 Hz and 10.4 Hz, CH_2_), 6.87 (t, 1H, *J* = 6.8 Hz, Ar-H), 6.94 (s, 1H, NH), 7.06 (t, 2H, *J* = 7.2 Hz, Ar-H), 7.17 (t, 4H, *J* = 8.0 Hz, Ar-H); 7.36 (d, 4H, *J* = 7.6 Hz, Ar-H), 7.47–7.58 (m, 3H, Ar-H), 7.76 (d, 1H, *J* = 8.4 Hz, Ar-H), 7.93 (d, 1H, *J* = 4.4 Hz, Ar-H), 8.02 (dt, 1H, *J* = 2.0 Hz and 5.6 Hz, Ar-H), 8.70 and 8.71 (dd, 1H, *J* = 1.2 Hz and 4.8 Hz, Ar-H), 11.11 (s, 1H, NH); ^13^C-NMR (DMSO-*d*_6_, 100 MHz) δ: 42.9, 46.1, 58.7, 87.3, 113.5, 119.9, 123.8, 125.3, 127.4, 128.7, 128.6, 138.3, 138.6, 142.2, 148.2, 149.8, 150.4, 150.6, 168.1, 170.4; IR (KBr, cm^−1^) ν_max_ = 3434, 3028, 1688, 1651, 1574, 1532, 1467, 1403., 1296, 1161, 757, 702, 555; [Anal. Calcd. for C_30_H_26_N_4_O_3_: C, 73.45; H, 5.34; N, 11.42; Found: C, 73.57; H, 5.46; N, 11.33]; LC/MS (ESI, *m/z*): [M+], found 490.20, C_30_H_26_N_4_O_3_ for 490.20.

*N*,*N*′-bis(5-Chloropyridin-2-yl)-4-oxo-2,6-di-*p*-tolylcyclohexane-1,1-dicarboxamide (**3b**). Yield 48 mg (0.09 mmol, 33%); m.p. 145–146 °C; ^1^H-NMR (DMSO-*d*_6_, 400 MHz) δ: 2.10 (s, 6H, CH_3_), 2.29–2.33 (m, 2H, CH_2_), 2.66 (t, 2H, *J* = 11.4 Hz, CH), 3.93 and 3.95 (dd, 2H, *J* = 4.8 Hz and 11.4 Hz, CH_2_), 6.96 (d, 4H, *J* = 8.0 Hz, Ar-H), 7.03 (s, 1H, NH), 7.18 (d, 4H, *J* = 8.0 Hz, Ar-H), 7.55 (d, 1H, *J* = 8.4 Hz, Ar-H), 7.70 and 7.72 (dd, 1H, *J* = 2.8 Hz and 9.2 Hz, Ar-H), 7.83 (d, 1H, *J* = 8.4 Hz, Ar-H), 8.01 (d, 1H, *J* = 2.0 Hz, Ar-H), 8.14 and 8.16 (dd, 1H, *J* = 2.8 Hz and 8.0 Hz, Ar-H), 8.76 (d, 1H, *J* = 2.4 Hz, Ar-H), 11.16 (s, 1H, NH); ^13^C-NMR (DMSO-*d*_6_, 100 MHz) δ: 20.9, 42.8, 45.7, 58.9, 87.6, 114.5, 125.6, 126.6, 128.5, 129.3, 130.9, 136.4, 138.2, 138.4, 139.0, 146.6, 148.3, 148.2, 149.2, 168.4, 170.7; IR (KBr, cm^−1^) ν_max_ = IR (KBr, cm^−1^) νmax = 3231, 3088, 1737, 1691, 1656, 1563, 1468, 1375, 1281, 1244, 1175, 1112, 1014, 828, 807, 682; [Anal. Calcd. for C_32_H_28_Cl_2_N_4_O_3_: C, 65.42; H, 4.80; N, 9.54; Found: C, 65.31; H, 4.93; N, 9.67; LC/MS (ESI, *m/z*): [M+], found 586.10; C_32_H_28_Cl_2_N_4_O_3_ for 586.15.

2,6-bis(4-Chlorophenyl)-4-oxo-*N*,*N*′-di(pyridin-2-yl)cyclohexane-1,1-dicarboxamide (**3c**). Yield 90 mg (0.16 mmol, 64%); m.p. 231–232 °C; ^1^H-NMR (DMSO-*d*_6_, 400 MHz) δ: 2.30 and 2.33 (dd, 2H, *J* = 5.6 Hz and 12.0 Hz, CH_2_), 2.69 (t, 2H, *J* = 12.4 Hz, CH), 4.00 and 4.02 (dd, 2H, *J* = 4.8 Hz and 10.4 Hz, CH_2_), 6.87 (t, 1H, *J* = 4.8 Hz, Ar-H), 7.01 (s, 1H, NH), 7.23 (d, 4H, *J* = 8.4 Hz, Ar-H); 7.37 (d, 4H, *J* = 8.4 Hz, Ar-H), 7.48–7.51 (m, 1H, Ar-H), 7.54 (d, 1H, *J* = 8.0 Hz, Ar-H), 7.60 (t, 1H, *J* = 7.2 Hz, Ar-H), 7.76 (d, 1H, *J* = 8.8 Hz, Ar-H), 7.98–8.03 (m, 2H, Ar-H), 8.69–8.70 (m, 1H, Ar-H), 11.11 (s, 1H, NH); ^13^C-NMR (DMSO-*d*_6_, 100 MHz) δ: 42.7, 45.3, 58.5, 87.2, 113.5, 120.2, 124.0, 125.5, 128.7, 130.6, 132.1, 138.5, 138.7, 141.0, 148.4, 149.8, 150.2, 150.4, 167.9, 170.2; IR (KBr, cm^−1^) ν_max_ = 3434, 3075, 1695, 1651, 1590, 1577, 1531, 1469, 1433, 1327, 1295, 1161, 1110, 1053, 991, 774, 555; [Anal. Calcd. for C_30_H_24_Cl_2_N_4_O_3_: C, 64.41; H, 4.32; N, 10.01; Found: C, 64.12; H, 4.53; N, 10.15]; LC/MS (ESI, *m/z*): [M+], found 558.10, C_30_H_24_Cl_2_N_4_O_3_ for 558.12.

2,6-bis(2,4-Dichlorophenyl)-4-oxo-*N*,*N*′-di(pyridin-2-yl)cyclohexane-1,1-dicarboxamide (**3d**). Yield 94 mg (0.15 mmol, 60%); m.p. 210–211 °C; ^1^H-NMR (DMSO-*d*_6_, 400 MHz) δ: 2.03 and 2.05 (dd, 2H, *J* = 5.2 Hz and 11.6 Hz, CH_2_), 2.89 (t, 2H, *J* = 11.6 Hz, CH), 4.53 and 4.55 (dd, 2H, *J* = 6.0 Hz and 11.2 Hz, CH_2_), 6.94–6.97 (m, 1H, Ar-H), 7.08 (s, 1H, NH), 7.37 (d, 1H, *J* = 7.6 Hz, Ar-H); 7.39 (d, 1H, *J* = 2.0 Hz, Ar-H), 7.41 (d, 1H, *J* = 2.0 Hz, Ar-H), 7.45 (d, 2H, *J* = 2.4 Hz, Ar-H), 7.47–7.50 (m, 1H, Ar-H), 7.58–7.62 (m, 1H, Ar-H), 7.68 (t, 3H, *J* = 8.4 Hz, Ar-H), 7.97 (dt, 1H, *J* = 2.0 Hz and 7.6, Ar-H), 8.05–8.06 (m, 1H, Ar-H), 8.65–8.67 (m, 1H, Ar-H), 11.00 (s, 1H, NH); ^13^C-NMR (DMSO-*d*_6_, 100 MHz) δ: 41.9, 42.8, 55.9, 86.9, 113.5, 120.3, 124.2, 125.4, 128.5, 129.3, 132.6, 134.6, 138.6, 138.8, 139.7, 148.4, 149.8, 149.9, 150.5, 166.9, 171.1; IR (KBr, cm^−1^) ν_max_ = 3435, 3076, 1694, 1650, 1589, 1573, 1530, 1467, 1435, 1329, 1296, 1163, 1111, 1050, 995, 820, 775, 569; [Anal. Calcd. for C_30_H_22_Cl_4_N_4_O_3_: C, 57.35; H, 3.53; N, 8.92; Found: C, 57.54; H, 3.67; N, 9.13]; LC/MS (ESI, *m/z*): [M+], found 626.10, C_30_H_22_Cl_4_N_4_O_3_ for 626.04.

2,6-bis(4-Bromorophenyl)-4-oxo-*N*,*N*′-di(pyridin-2-yl)cyclohexane-1,1-dicarboxamide (**3e**). Yield 90 mg (0.14 mmol, 56%); m.p. 227–228 °C; ^1^H-NMR (DMSO-*d*_6_, 400 MHz) δ: 2.31 and 2.34 (dd, 2H, *J* = 4.4 Hz and 12.0 Hz, CH_2_), 2.69 (t, 2H, *J* = 12.4 Hz, CH), 4.00 and 4.02 (dd, 2H, *J* = 4.4 Hz and 10.8 Hz, CH_2_), 6.93 (t, 1H, *J* = 6.0 Hz, Ar-H), 7.00 (s, 1H, NH), 7.31 (d, 4H, *J* = 8.4 Hz, Ar-H); 7.39 (d, 4H, *J* = 8.4 Hz, Ar-H), 7.49 (t, 1H, *J* = 6.0 Hz, Ar-H), 7.52 (d, 1H, *J* = 8.0 Hz, ArH), 7.61 (t, 1H, *J* = 8.0 Hz, Ar-H), 7.78 (d, 1H, *J* = 8.0 Hz, Ar-H), 7.91–8.03 (m, 2H, Ar-H), 8.69 and 8.70 (dd, 1H, *J* = 1.2 Hz and 3.6 Hz, Ar-H), 11.11 (s, 1H, NH); ^13^C-NMR (DMSO-*d*_6_, 100 MHz) δ: 42.6, 45.4, 58.4, 87.2, 113.5, 120.2, 120.7, 124.0, 125.5, 130.9, 131.7, 138.5, 138.7, 141.4, 148.4, 149.8, 150.2, 150.4, 167.9, 170.2; IR (KBr, cm^−1^) ν_max_ = 3414, 3055, 1687, 1651, 1589, 1574, 1531, 1487, 1467, 1435, 1402, 1297, 1158, 1071, 1010, 993, 838, 819, 773, 554; [Anal. Calcd. for C_30_H_24_Br_2_N_4_O_3_: C, 55.58; H, 3.73; N, 8.64; Found: C, 55.71; H, 3.86; N, 8.53]; LC/MS (ESI, *m/z*): [M+], found 646.00 C_30_H_24_Br_2_N_4_O_3_ for 646.02.

*N*,*N*′-bis(5-Chloropyridin-2-yl)-2,6-bis(3-nitrophenyl)-4-oxocyclohexane-1,1-dicarboxamide (**3f**). Yield 100 mg (0.15 mmol, 62%); m.p. 174–175 °C; ^1^H-NMR (DMSO-*d*_6_, 400 MHz) δ: 2.29 and 2.32 (dd, 2H, *J* = 5.6 Hz and 12.8 Hz, CH_2_), 2.74 (t, 1H, *J* = 513.2 Hz, CH), 3.02 (t, 1H, *J* = 13.2 Hz, CH), 4.06 and 4.09 (dd, 1H, *J* = 6.0 Hz and 10.1 Hz, CH_2_), 4.28 and 4.31 (dd, 1H, *J* = 6.4 Hz and 10.1 Hz, CH_2_), 7.17 (s, 1H, NH), 7.33 (d, 1H, *J* = 8.8 Hz, Ar-H), 7.47 (t, 1H, *J* = 7.6 Hz, Ar-H), 7.55 (d, 1H, *J* = 8.4 Hz, Ar-H), 7.66–7.70 (m, 2H, Ar-H), 7.75 (d, 1H, *J* = 8.0 Hz, Ar-H), 7.94 and 7.96 (dd, 1H, *J* = 2.0 Hz and 8.0 Hz, Ar-H), 8.02 (d, 2H, *J* = 7.6 Hz, Ar-H), 8.15–8.20 (m, 3H, Ar-H), 8.27 (d, 1H, *J* = 2.4 Hz, Ar-H), 8.70 (d, 1H, *J* = 2.4 Hz, Ar-H), 10.78 (s, 1H, NH); ^13^C-NMR (DMSO-*d*_6_, 100 MHz) δ: 43.9, 44.6, 59.1, 87.4, 114.9, 122.0, 123.0, 124.4, 124.5, 126.0, 126.7, 130.0, 130.4, 131.0, 133.7, 135.9, 138.2, 138.4, 141.5, 145.8, 146.9, 147.8, 148.0, 148.1, 148.8, 149.6, 158.4, 168.5, 170.7; IR (KBr, cm^−1^) ν_max_ = 3236, 3087, 1733, 1693, 1650, 1568, 1528, 1463, 1374, 1351, 1286, 1240, 1170, 1114, 1015, 826, 806, 686; [Anal. Calcd. for C_30_H_22_Cl_2_N_6_O_7_: C, 55.48; H, 3.41; N, 12.94; Found: C, 55.62; H, 3.54; N, 13.08; LC/MS (ESI, *m/z*): [M+], found 648.10; C_30_H_22_Cl_2_N_6_O_7_ for 648.09.

*N*,*N*′-bis(5-Chloropyridin-2-yl)-2,6-bis(4-methoxyphenyl)-4-oxocyclohexane-1,1-dicarboxamide (**3g**). Yield 110 mg (0.18 mmol, 72%); m.p. 203–204 °C; ^1^H-NMR (DMSO-*d*_6_, 400 MHz) δ: 2.30 and 2.33 (dd, 2H, *J* = 5.2 Hz and 11.6 Hz, CH_2_), 2.66 (t, 2H, *J* = 11.6 Hz, CH), 3.58 (s, 6H, OCH_3_), 3.91 and 3.94 (dd, 2H, *J* = 4.8 Hz and 11.6 Hz, CH_2_), 6.72 (d, 4H, *J* = 9.6 Hz, Ar-H), 7.02 (s, 1H, NH), 7.22 (d, 4H, *J* = 9.6 Hz, Ar-H), 7.57 (d, 1H, *J* = 9.6 Hz, Ar-H), 7.71 and 7.73 (dd, 1H, *J* = 2.4 Hz and 8.8 Hz, Ar-H), 7.84 (d, 1H, *J* = 9.2 Hz, Ar-H), 8.02 (d, 1H, *J* = 2.8 Hz, Ar-H), 8.13 and 8.15 (dd, 1H, *J* = 2.8 Hz and 8.0 Hz, Ar-H), 8.77 (d, 1H, *J* = 2.4 Hz, Ar-H), 11.16 (s, 1H, NH); ^13^C-NMR (DMSO-*d*_6_, 100 MHz) δ: 40.5, 45.2, 55.3, 59.3, 87.6, 114.1, 114.6, 125.6, 126.7, 129.7, 130.9, 133.9, 138.2, 138.3, 146.6, 148.3, 148.8, 149.2, 158.4, 168.5, 170.7; IR (KBr, cm^−1^) ν_max_ = 3124, 2960, 2833, 1693, 1656, 1610, 1569, 1512, 1460, 1409, 1376, 1305, 1251, 1177, 1156, 1107, 1031, 1015, 837, 574; [Anal. Calcd. for C_32_H_28_Cl_2_N_4_O_5_: C, 62.04; H, 4.56; N, 9.04; Found: C, 61.87; H, 4.45; N, 9.19; LC/MS (ESI, *m/z*): [M+], found 618.14 C_32_H_28_Cl_2_N_4_O_5_ for 618.14.

*N*,*N*′-bis(5-Chloropyridin-2-yl)-2,6-di(naphthalen-2-yl)-4-oxocyclohexane-1,1-dicarboxamide (**3h**). Yield 95 mg (0.14 mmol, 58%); m.p. 114–115 °C; ^1^H-NMR (DMSO-*d*_6_, 400 MHz) δ: 2.22–2.27 (m, 2H, CH_2_), 3.07 (t, 2H, *J* = 12.0 Hz, CH), 5.24–5.27 (m, 2H, CH_2_), 6.80 (d, 1H, *J* = 9.2 Hz, Ar-H), 7.07 (s, 1H, NH), 7.26 and 7.28 (dd, 1H, *J* = 2.4 Hz and 8.8 Hz, Ar-H), 7.39–7.47 (m, 5H, Ar-H), 7.58 (t, 2H, *J* = 8.0 Hz, Ar-H), 7.66 (d, 2H, *J* = 8.4 Hz, Ar-H), 7.72 (d, 2H, *J* = 8.4 Hz, Ar-H), 7.76 (d, 2H, *J* = 8.4 Hz, Ar-H), 7.92 (d, 1H, *J* = 2.8 Hz, Ar-H), 8.10 and 8.12 (dd, 1H, *J* = 2.8 Hz and 8.8 Hz, Ar-H), 8.47 (d, 2H, *J* = 8.4 Hz, Ar-H), 8.77 (d, 1H, *J* = 2.8 Hz, Ar-H), 11.13 (s, 1H, NH); ^13^C-NMR (DMSO-*d*_6_, 100 MHz) δ: 40.7(merged with dmso-*d*_6_), 44.4, 57.0, 87.5, 114.0, 123.5, 124.5, 125.3, 126.0, 126.1, 126.3, 126.6, 127.6, 128.8, 131.1, 131.7, 133.7, 137.5, 138.5, 140.2, 146.4, 148.4, 148.8, 148.9, 168.1, 172.3; IR (KBr, cm^−1^) ν_max_ = 3234, 3085, 1737, 1692, 1654, 1569, 1521, 1464, 1377, 1359, 1287, 1248, 1173, 1113, 1018, 824, 807; [Anal. Calcd. for C_38_H_28_Cl_2_N_4_O_3_: C, 69.20; H, 4.28; N, 8.49; Found: C, 69.11; H, 4.19; N, 8.67; LC/MS (ESI, *m/z*): [M+], found 658.10; C_38_H_28_Cl_2_N_4_O_3_ for 658.15.

*N*,*N*′-bis(5-Chloropyridin-2-yl)-4-oxo-2,6-di(thiophen-2-yl)cyclohexane-1,1-dicarboxamide (**3i**). Yield 85 mg (0.15 mmol, 60%); m.p. 165–166 °C; ^1^H-NMR (DMSO-*d*_6_, 400 MHz) δ: 2.36 (d, 2H, *J* = 11.6 Hz, CH_2_), 2.75 (t, 2H, *J* = 11.6 Hz, CH), 4.26 and 4.28 (m, 2H, *J* = 4.4 Hz and 10.0 Hz, CH_2_), 6.83 (t, 2H, *J* = 4.0 Hz, Ar-H), 6.99 (d, 2H, *J* = 2.4 Hz, Ar-H), 7.13 (s, 1H, NH), 7.24 (d, 2H, *J* = 4.4 Hz, Ar-H), 7.48 (d, 1H, *J* = 8.4 Hz, Ar-H), 7.80 (d, 1H, *J* = 11.2 Hz, Ar-H), 7.99 (d, 1H, *J* = 8.4 Hz, Ar-H), 8.09 (s, 1H, Ar-H), 8.13 (d, 1H, *J* = 7.2 Hz, Ar-H), 8.73 (s, 1H, Ar-H), 11.31 (s, 1H, NH); ^13^C-NMR (DMSO-*d*_6_, 100 MHz) δ: 40.5 (merged with dmso-*d*_6_), 40.5, 43.9, 59.9, 87.4, 114.8, 125.5, 125.9, 126.1, 126.5, 127.4, 131.0, 138.3, 138.4, 144.7, 146.8, 148.3, 148.6, 149.3, 168.5, 170.0; IR (KBr, cm^−1^) ν_max_ = 3426, 3236, 2951, 2925, 1687, 1569, 1530, 1461, 1374, 1291, 1155, 1111, 1015, 851, 835, 696, 582; [Anal. Calcd. for C_26_H_20_Cl_2_N_4_O_3_S_2_: C, 54.64; H, 3.53; N, 9.80; Found: C, 54.72; H, 3.41; N, 9.97; LC/MS (ESI, *m/z*): [M+], found 570.00; C_26_H_20_Cl_2_N_4_O_3_S_2_ for 570.04. 

*N*,*N*′-bis(5-Chloropyridin-2-yl)-2,6-di(furan-2-yl)-4-oxocyclohexane-1,1-dicarboxamide (**3j**). Yield 70 mg (0.13 mmol, 52.0%); m.p. 160–161 °C; ^1^H-NMR (DMSO-*d*_6_, 400 MHz) δ: 2.27 and 2.29 (dd, 2H, *J* = 4.4 Hz and 11.2 Hz, CH_2_), 2.57 (t, 2H, *J* = 11.2 Hz, CH), 4.07 and 4.10 (dd, 2H, *J* = 4.8 Hz and 10.8 Hz, CH_2_), 6.20 (d, 2H, *J* = 2.8 Hz, Ar-H), 6.23–6.24 (m, 2H, Ar-H), 7.07 (s, 1H, NH), 7.37 (d, 1H, *J* = 8.4 Hz, Ar-H), 7.40 (s, 2H, Ar-H), 7.81 and 7.83 (dd, 1H, *J* = 2.8 Hz and 8.8 Hz, Ar-H), 8.01 (d, 1H, *J* = 8.4 Hz, Ar-H), 8.05 and 8.08 (dd, 1H, *J* = 2.4 Hz and 8.4 Hz, Ar-H), 8.15 (d, 1H, *J* = 2.8 Hz, Ar-H), 8.67 (d, 1H, *J* = 2.8 Hz, Ar-H), 11.40 (s, 1H, NH); ^13^C-NMR (DMSO-*d*_6_, 100 MHz) δ: 38.7, 39.3, 40.6, 55.9, 87.1, 107.3, 110.9, 114.8, 125.8, 126.4, 130.8, 138.2, 138.4, 143.0, 146.8, 148.0, 148.6, 149.6, 154.6, 168.3, 169.9; IR (KBr, cm^−1^) ν_max_ = 3420, 3243, 2952, 1691, 1630, 1569, 1530, 1463, 1418, 1375, 1291, 1163, 1112, 1014, 809, 735; [Anal. Calcd. for C_26_H_20_Cl_2_N_4_O_5_: C, 57.90; H, 3.74; N, 13.15; Found: C, 58.11; H, 3.63; N, 12.89; LC/MS (ESI, *m/z*): [M+], found 538.1; C_26_H_20_Cl_2_N_4_O_5_ for 538.08.

2,6-bis(3-Bromophenyl)-*N*,*N*′-bis(5-chloropyridin-2-yl)-4-oxocyclohexane-1,1-dicarboxamide (**3k**). Yield 78 mg (0.11 mmol, 44.0%); m.p. 213–214 °C; ^1^H-NMR (DMSO-*d*_6_, 400 MHz) δ: 2.32 and 2.35 (dd, 2H, *J* = 4.8 Hz and 11.6 Hz, CH_2_), 2.69 (t, 2H, *J* = 11.2 Hz, CH), 4.00 and 4.04 (dd, 2H, *J* = 5.2 Hz and 10.8 Hz, CH_2_), 7.15 (s, 1H, NH), 7.17 (t, 2H, *J* = 8.0 Hz, Ar-H), 7.30 (t, 4H, *J* = 8.0 Hz, Ar-H), 7.53 (s, 2H, Ar-H), 7.56 (d, 1H, *J* = 8.0 Hz, Ar-H), 7.76 and 7.78 (dd, 1H, *J* = 2.0 Hz and 8.8 Hz, Ar-H), 7.83 (d, 1H, *J* = 8.0 Hz, Ar-H), 8.05 (d, 1H, *J* = 2.8 Hz, Ar-H), 8.18 and 8.20 (dd, 1H, *J* = 2.8 Hz and 8.0 Hz, Ar-H), 8.74 (d, 1H, *J* = 2.4 Hz, Ar-H), 11.09 (s, 1H, NH); ^13^C-NMR (DMSO-*d*_6_, 100 MHz) δ: 42.2, 45.4, 58.8, 87.6, 114.5, 121.9, 126.0, 126.6, 127.4, 130.5, 131.0, 131.2, 131.8, 138.3, 138.6, 144.3, 146.8, 148.2, 148.6, 148.8, 167.9, 170.3; IR (KBr, cm^−1^) ν_max_ = 3386, 3249, 2930, 1688, 1657, 1569, 1519, 1460, 1372, 1305, 1286, 1153, 1114, 1009, 834, 804, 696; [Anal. Calcd. for C_30_H_22_Br_2_Cl_2_N_4_O_3_: C, 50.24; H, 3.09; N, 7.81; Found: C, 50.07; H, 3.26; N, 7.92; LC/MS (ESI, *m/z*): [M+], found 714.00; C_30_H_22_Br_2_Cl_2_N_4_O_3_ for 713.94.

*N*,*N*′-bis(5-Chloropyridin-2-yl)-4-oxo-2,6-bis(4-(trifluoromethyl)phenyl)cyclohex-ane-1,1-dicarboxamide (**3l**). Yield 64 mg (0.92 mmol, 37.0%); m.p. 138–139 °C; ^1^H-NMR (DMSO-*d*_6_, 400 MHz) δ: 2.36 and 2.39 (dd, 2H, *J* = 4.4 Hz and 11.6 Hz, CH_2_), 2.75 (t, 2H, *J* = 11.2 Hz, CH), 4.14 and 4.16 (dd, 2H, *J* = 5.2 Hz and 10.8 Hz, CH_2_), 7.23 (s, 1H, NH), 7.53–7.57 (m, 9H, Ar-H), 7.67 (d, 1H, *J* = 8.8 Hz, Ar-H), 7.72–7.43 (m, 1H, Ar-H), 7.98–7.99 (m, 1H, Ar-H), 8.15 and 8.18 (dd, 1H, *J* = 2.8 Hz and 8.4 Hz, Ar-H), 8.77 (d, 1H, *J* = 2.8 Hz, Ar-H), 10.99 (s, 1H, NH); ^13^C-NMR (DMSO-*d*_6_, 100 MHz) δ: 42.0, 45.8, 58.6, 87.6, 114.3, 123.1, 125.7, 125.9, 126.9, 128.0, 128.4, 129.5, 131.2, 138.2, 138.5, 146.4, 146.7, 148.4, 148.5, 148.7, 167.7, 170.2; IR (KBr, cm^−1^) ν_max_ = 3195, 2956, 1693, 1653, 1570, 1523, 1463, 1399, 1376, 1324, 1163, 1124, 1068, 1017, 841, 609; [Anal. Calcd. for C_32_H_22_Cl_2_F_6_N_4_O_3_: C, 55.27; H, 3.19; N, 8.06; Found: C, 55.13; H, 3.39; N, 8.17; LC/MS (ESI, *m/z*): [M+], found 694.10; C_32_H_22_Cl_2_F_6_N_4_O_3_ for 694.10.

2,6-bis(4-Fluorophenyl)-4-oxo-*N*,*N*′-di(pyridin-2-yl)cyclohexane-1,1-dicarboxamide (**3m**). Yield 112 mg (0.21 mmol, 85%); m.p. 245–246 °C; ^1^H-NMR (DMSO-*d*_6_, 400 MHz) δ: 2.32 and 2.35 (dd, 2H, *J* = 4.4 Hz and 11.2 Hz, CH_2_), 2.69 (t, 2H, *J* = 12.0 Hz, CH), 4.07 and 4.04 (m, 2H, CH_2_), 6.89–6.92 (m, 1H, Ar-H), 6.93 (s, 1H, NH), 7.02 (t, 4H, *J* = 9.2 Hz, Ar-H), 7.37–7.41 (m, 4H, Ar-H), 7.48–7.51 (m, 1H, Ar-H), 7.53–7.56 (m, 1H, ArH), 7.57–7.61 (m, 1H, Ar-H), 7.76–7.78 (m, 1H, Ar-H), 7.96–7.98 (m, 1H, Ar-H), 8.01 (dt, 1H, *J* = 2.0 Hz and 8.0 Hz, Ar-H), 8.69–8.71 (m, 1H, Ar-H), 11.11 (s, 1H, NH); ^13^C-NMR (DMSO-*d*_6_, 100 MHz) δ: 42.9, 45.2, 58.8, 87.2, 113.5, 115.4, 115.6, 120.1, 123.9, 125.5, 130.6, 130.7, 138.2, 138.3, 138.4, 138.6, 148.3, 149.8, 150.3, 150.5, 160.3, 162.7, 168.1, 170.3; IR (KBr, cm^−1^) ν_max_ = 3421, 3065, 1678, 1655, 1589, 1576, 1533, 1488, 1462, 1434, 1408, 1291, 1154, 1077, 1011, 993, 839, 815, 770, 559; [Anal. Calcd. for C_30_H_24_F_2_N_4_O_3_: C, 68.43; H, 4.59; N, 10.64; Found: C, 68.57; H, 4.71; N, 10.42]; LC/MS (ESI, *m/z*): [M+], found 526.20 C_30_H_24_F_2_N_4_O_3_ for 526.18.

### 3.2. Anticancer Activity 

#### 3.2.1. Cell Lines and Drugs 

The cytotoxic activity of the new synthesized compounds was tested in different mammalian cancer cells, breast cancer (+ve ER) (MCF-7), breast cancer (−ve ER) (MDA-MB-231), tongue (oral cancer) (SAS), prostate cancer (PC-3), colorectal cancer (HCT-116) and hepatocellular carcinoma (HuH-7 and HepG-2). The cell lines were obtained from the American Type Culture Collection (ATCC). The cells were cultivated at 37 °C and 5% CO_2_ in DMEM (Lonza) medium supplemented with 10% fetal bovine serum (Lonza), 100 IU/mLpenicillin and 100 µg/mL streptomycin (Lonza). Cisplatin was used as a positive control and was obtained from Sigma-Aldrich. The synthesized compounds were solubilized in DMSO and stored at −20 °C. For the initial screening, 0.5% crystal violet was used [21]. The viability of the cells were determined by using the MTT reagent [22,23].

#### 3.2.2. Cytotoxicity Assay 

“The cells were seeded in a 96-well plate and serial dilutions of the tested compounds or cisplatin was added after overnight incubation of the cells at 37 °C and 5% CO_2_. DMSO was used as a negative control (0.1%). After that, MTT (5 mg/mL PBS) was added after 48 hours of incubation. The formazan crystals were solubilized by the acidified SDS solution. The absorbance was recorded at 570 nm by Biotech ELx-800™ plate reader (Winooski, VT, USA). The viability assay was performed 3 times and the standard deviation was determined (±). IC_50_ was calculated as the concentration that causes 50% inhibition of cell growth. The selectivity index was calculated as previously reported” [26,27].

#### 3.2.3. α-Glucosidase Inhibitory Assay

“Certain aliquots (40 μL) of compounds (prepared in 50% DMSO and 50% water) at different concentrations (3–500 μg/mL) were pre-incubated with a potassium phosphate buffer (80 μL, pH 6.8), containing 67 mM potassium phosphate and 2.0 unit/ml α-glucosidase in a 96-well plate for 10 min. After that, 40 μL of 5 mM p-nitrophenyl-α-d-glucopyranoside solution (p-NPG) in potassium phosphate buffer was added into the mixture and incubated for another 10 min. After incubation, 100 mM Na_2_CO_3_ (60 μL) was added into the mixture to terminate the reaction and the absorbance of the mixture was measured at a wavelength of 415 nm. The experiment was also carried out using a standard inhibitor, namely acarbose (positive control). The concentration resulting in 50% inhibition of α-glucosidase activity (IC_50_) was determined by using GraphPad Prism 5 statistical package (GraphPad^®^ Software Inc., San Diego, CA, USA). All data were expressed as means ± standard deviations of triplicate determinations” [28].

#### 3.2.4. Molecular Docking Study 

The docking studies were performed using the OpenEye Modeling software (License 2018-2019, OpenEye Scientific, NM, USA) [29,30,31]. A virtual library of the target compounds was used and their energies were minimized using the MMFF94 force field, followed by the generation of multi-conformers using the OMEGA application. The whole library of minimized energy values was used to dock an appropriate target according to the reported crystalized standard. The receptor PDB files for EGFR were downloaded from the Protein Data Bank (PDB:ID: 1M17). Both the ligand input file and the receptor input file were used as the input into FRED to perform the molecular docking simulations. Multiple scoring functions were employed to predict the energy profile of the ligand–receptor complex. The VIDA application was employed as a visualization tool to show the pose of the ligands and the potential binding interactions of the ligands to the receptor of interest.

## 4. Conclusions

The present study mainly focuses on the synthesis of a new series of pyridine-dicarboximide-cyclohexanone-based chemical entities with improved anticancer activities. This new series was obtained via the DBU basic system, which exerts significant effects by promoting the Michael addition reaction. The synthesized compounds were screened against different cancer cell lines and were evaluated for their α-glucosidase inhibitory activities. Consequently, the compounds **3c**, **3e** and **3l** showed the most promising anticancer activities against different cancer cell lines. Thus, further studies are warranted to evaluate the underlying mechanism.

## Figures and Tables

**Table 1:** Procedure for the synthesis of the target compound **3a**–**m**.

#	R	2a–m	Ar	Product 3a–m	Yield, %
1	H	**2a**	C_6_H_5_	**3a**	89
2	Cl	**2b**	*p*-CH_3_C_6_H_4_	**3b**	33
3	H	**2c**	*p*-ClC_6_H_4_	**3c**	64
4	H	**2d**	2,4-Cl_2_C_6_H_3_	**3d**	60
5	H	**2e**	*p*-BrC_6_H_4_	**3e**	56
6	Cl	**2f**	*m*-NO_2_C_6_H_4_	**3f**	62
7	Cl	**2g**	*p*-MeOC_6_H_4_	**3g**	72
8	Cl	**2h**	β-Naphthalene	**3h**	58
9	Cl	**2i**	2-Thiophene	**3i**	60
10	Cl	**2j**	2-Furan	**3j**	52
11	Cl	**2k**	*m*-BrC_6_H_4_	**3k**	44
12	Cl	**2l**	*p*-CF_3_C_6_H_4_	**3l**	37
13	H	**2m**	*p*-FC_6_H_4_	**3m**	85

**Table 2:** The cytotoxic activities of the test compounds against seven cancer cell lines representing five different types of cancers (breast, tongue, prostate, colon and liver). Cell viability was evaluated with the MTT assay and the IC_50_ (µM) value was calculated. The values are represented as the mean ± standard deviation from three independent experiments. NA indicates that the compounds were not active during the initial screening of their anticancer activities using crystal violet assay.

Compounds ^a,b^	Breast		Oral	Prostate	Colon	Liver	
	MCF-7	MDA-MB-231	SAS	PC-3	HCT-116	HuH-7	HepG2
**3a**	NA ^c^	NA	NA	NA	NA	NA	NA
**3b**	NA	NA	NA	NA	NA	NA	NA
**3c**	10 ± 0.62	7 ± 1.12	15 ± 1.3	25 ± 1.42	NA	NA	8 ± 0.89
**3d**	>50 ± 1.17	18 ± 0.87	NA	NA	NA	NA	> 50
**3e**	12 ± 0.54	5 ± 0.5	NA	NA	NA	NA	8 ± 0.96
**3f**	NA	NA	NA	NA	NA	NA	NA
**3g**	NA	NA	NA	NA	NA	NA	NA
**3h**	NA	NA	NA	NA	NA	NA	NA
**3i**	NA	NA	NA	NA	NA	NA	NA
**3j**	50 ± 0.78	45±3	NA	NA	NA	NA	>50 ± 1.08
**3k**	NA	NA	NA	NA	NA	NA	NA
**3l**	18 ± 1.71	5 ± 0.25	9 ± 0.38	> 50 ± 2	6 ± 0.78	4.5 ± 0.3	25 ± 0.38
**3m**	NA	NA	NA	NA	NA	NA	NA
**Cisplatin**	9 ± 2.43	15 ± 0.71	4.5 ± 0.34	12 ± 1.25	8 ± 0.76	14.7 ± 0.5	10 ± 0.65

^a^ All test compounds showed a value of IC_50_ > 100 µM against all seven cell lines (very high safety margin); ^b^ In comparison with cisplatin IC_50_ (µM) value, which was very marginal and in the range of 15–20 µM for MCF-7, MDA-MB-231, SAS, PC-3, HCT-11, HuH-7 and HepG2; ^c^ NA, no or negligible activity.

**Table 3:** Results of α-glucosidase inhibitory activity.

Compounds	IC_50_ (± SEM μmol/L)
**3d**	148.18 ± 3.02
**3i**	418.21 ± 1.02
**3j**	124.24 ± 0.16
**Acarbose**	32.71 ± 1.17
